# First genetic characterization of *Xeroderma pigmentosum* in Libya: High frequency of XP‐C founder mutation

**DOI:** 10.1002/mgg3.2158

**Published:** 2023-02-22

**Authors:** Najlaa Khalat, Olfa Messaoud, Mariem Ben Rekaya, Mariem Chargui, Mohamed Zghal, Bashir Zendah, Najat Saqer, Mourad Mokni, Sonia Abdelhak, Othman A. Mohamed

**Affiliations:** ^1^ The Libyan Academy Tripoli Libya; ^2^ Institut Pasteur de Tunis, Biomedical Genomics and Oncogenetics Laboratory University Tunis El Manar Tunis Tunisia; ^3^ Dermatology Department Charles Nicolle Hospital Tunis Tunisia; ^4^ Dermatology Department Medical Tripoli Centre (MTC) Tripoli Libya; ^5^ Dermatology Department Central Tripoli Hospital Tripoli Libya; ^6^ Dermatology Department La Rabta Hospital Tunis Tunisia; ^7^ Present address: Reproduction, Maternal and Child Health Laboratory CHU of Quebec‐Laval University Quebec City Quebec Canada; ^8^ Present address: Biotechnology Research Centre Tripoli Libya

**Keywords:** founder effect, Maghreb mutations, mutational heterogeneity, *Xeroderma pigmentosum*, Libya

## Abstract

**Background:**

*Xeroderma pigmentosum* is an autosomal recessive disease characterized by a high sensitivity to UV radiations. The disease is clinically and genetically heterogeneous, thus making accurate early clinical diagnosis difficult. Although the disease is considered rare worldwide, previous studies have shown that it is more frequent in Maghreb countries. So far, no genetic study has been published on Libyan patients, except three reports limited to clinical descriptions.

**Methods:**

Our study, which represents the first genetic characterization of XP in Libya, was conducted on 14 unrelated families including 23 Libyan XP patients with a consanguinity rate of 93%. Blood samples were collected from 201 individuals including patients and their relatives. Patients were screened for founder mutations already described in Tunisia.

**Results:**

The two founder Maghreb XP mutations, XPA p.Arg228* associated with the neurological form and XPC p.Val548Alafs*25 in patients with only cutaneous manifestations, were homozygously identified. The latter was predominant (19 of 23 patients). In addition, another XPC homozygous mutation (p.Arg220*) has been identified in only one patient. For the remaining patient, the absence of founder *XPA*, *XPC*, *XPD,* and *XPG* mutations suggests mutational heterogeneity of XP in Libya.

**Conclusion:**

Identification of common mutations with other Maghreb populations is in favor of a common ancestor in North‐African populations.

## Graphical abstract

This is the first genetic characterization of XP in Libya that was carried out on 23 Libyan XP patients with a consanguinity rate of 93%. The study showed the presence of common mutations with other Maghreb populations which is in favor of a common ancestor in North‐African populations. Our findings represent a valuable tool for genetic counseling and prenatal diagnosis for this incurable disease.

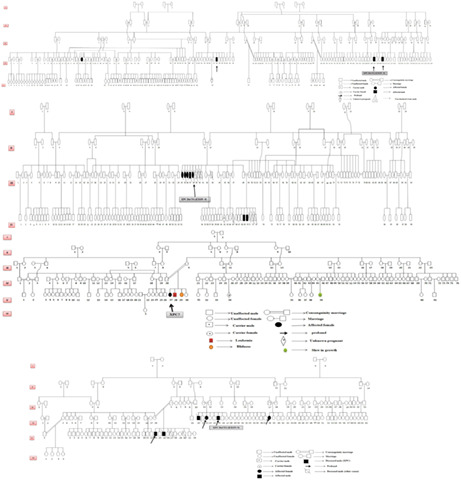

## 1 INTRODUCTION

*Xeroderma pigmentosum* (XP, OMIM 278700–278780) is a rare autosomal recessive disease with a high clinical and genetic heterogeneity. *Xeroderma pigmentosum* patients exhibit extreme cutaneous and ocular photosensitivity associated with a high cutaneous malignancy predisposition. Some patients suffer from neurological alterations besides cutaneous lesions. Patient's photosensitivity results from their incapacity to repair UV‐induced DNA lesions via the Nucleotide Excision Repair (NER) or the Translesion Synthesis (TLS) pathway. Seven genes (*XPA* to *XPG*) are known to be responsible for XP related to NER deficiency while only one gene, *POLH* (OMIM 603968), is involved in XP via the TLS replication way.

*Xeroderma pigmentosum* is highly heterogeneous, both at clinical and at genetic levels. Its prevalence is also variable according to the geographical origin. Indeed, XP prevalence is of 1/1,000,000 in USA and Europe (Kleijer et al., 2008), 1/20,000–100,000 in Japan (Hirai et al., 2006; Moriwaki & Kraemer, 2001), and reaches 1/10,000–30,000 in North Africa (Fazaa et al., 2001; Khatri et al., 1999; Moussaid et al., 2004; Zghal et al., 2005). The disease has been reported in several regions and cities in Libya, through the registry database in dermatology departments of several hospitals across the country (Khatri et al., 1992, 1999; Visweswara et al., 1997). The prevalence of this disorder among the Libyan population is not known, but it is expected to be higher than its prevalence in other parts of the world because of the consanguinity and endogamy that are culturally favored.

Previous studies in populations from North African countries have shown the predominance of two complementation groups, XP‐C (OMIM 613208) and XP‐A (OMIM 611153) with common founder mutations. Taking into account the high frequency of XP in the region associated with its poor prognosis with the absence of any efficient therapy, genetic diagnosis is crucial for genetic counseling and prenatal diagnosis implementation. For this, we have conducted this study, which, to the best of our knowledge, represents the first genetic characterization of XP in Libya.

## 2 PATIENTS AND METHODS

### 2.1 Patients

Twenty‐three XP patients belonging to 14 unrelated families with a rate of consanguinity of 93% were clinically diagnosed by dermatology specialists in the hospitals (Tripoli Central Hospital and Tripoli Medical Center) in Libya and/or the Dermatology Department of Habib Thameur Hospital and La Rabta hospital in Tunis. Apart from one family that originated from the North‐East, all XP patients originated from cities located in the North‐Western Region of Libya.

Diagnosis of XP was suspected on the basis of typical clinical features such as photosensitivity, photophobia, and the presence of skin cancers. All patients and their parents were informed of the aims of this study and gave informed consent to the genetic analysis. A specific questionnaire has been elaborated in order to collect genealogical and clinical data and to identify other possible XP cases among their relatives.

### 2.2 Mutation analysis

**FIGURE 1:**
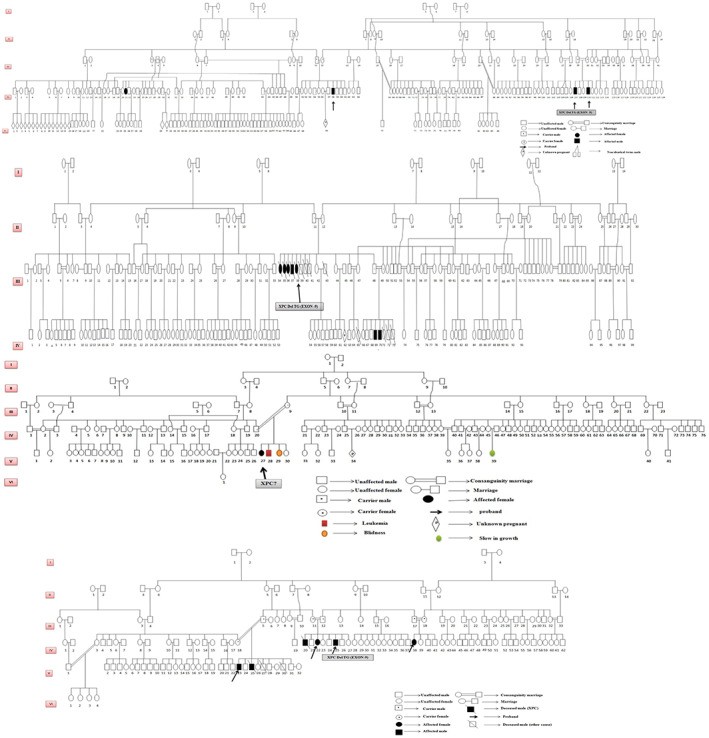
Examples of large pedigrees showing high connection and informativeness among XP families.

Blood samples were collected from XP patients and their family members whenever available. Taking into consideration the high relatedness and close connection that still exist between families in Libya, large pedigrees were drawn (Figure 1) and blood samples were collected from 201 individuals involving patients and their relatives. Genomic DNA was extracted from white blood cells by PrepFiler®ExpressForensic DNA Extraction Kit except for five patients for whom extraction was performed by salting out method. Several coding exons of *XP* genes and their exon–intron boundaries were amplified before being sequenced with the Big Dye terminator kit (Applied Biosystems, Foster City, CA, USA) using PCR primers on an ABI prism 3130 DNA Genetic Analyzer (Applied Biosystems) in accordance with the manufacturer's recommendations.

Mutations were annotated by Mutalyzer (https://mutalyzer.nl) according to HGVS nomenclature.

## 3 RESULTS

In this study, we report on genetic investigation of XP for 14 Libyan families including 23 patients with a sex ratio of 0.64. Their age ranges from one year and 9 months to 35 years. All patients showed typical clinical features of XP (Table 1). These include the presence of photophobia and photosensitivity. The photosensitivity is characterized by persistent late‐onset erythema followed by dyschromia. Photosensitivity is present in XP‐A, and XP‐C patients. However, it is less marked in XP‐C, and it requires prolonged exposure to manifest. The neurological status in patients belonging to family XPA94 is essentially marked by hyporeflexia.

**TABLE 1:** Characteristics of the Libyan XP patients.

Family code	XP group	Consanguinity	Familial history	Geographical origin	Individual code	Sex F/M	Age at study (years)	Age at onset^a^ (months)	Clinical diagnosis			Mutation	HM/HZ
									P.S	P.P	S.C		
XPA94	XP‐A	Yes	Yes	Al Bayda	**XPA94‐2**	M	18	ND	+++	+++	>3 SCC + 1 Melanoma	p.Arg228*	HM
					**XPA94‐3**	M	21	ND	+	+	3 SCC	p.Arg228*	HM
XPC127	XP‐C	Yes	No	Tripoli	**XPC127‐3**	M	1y 9 m	8	+	+	−	p.Val548Alafs*25	HM
XP166	XP‐?	Yes	No	Benghazi	**XPA166‐1**	F	5	1	+	+	−	No mutation detected	−
XP‐LIBYA‐1	XP‐C	Yes	Yes	Souq El‐Khamees	**LI‐117**	F	2	18	++	−	−	p.Val548Alafs*25	HM
XP‐LIBYA‐2	XP‐C	No	No	Al‐Khums	**LI‐208**	M	9	12	−	+	−	p.Val548Alafs*25	HM
XP‐LIBYA‐3	XP‐C	Yes	Yes	Souq EL‐Khamees	**LI‐192**	M	20	24	++	+++	Skin tumors	p.Val548Alafs*25	HM
					**LI‐111**	M	5	24	+	−	−	p.Val548Alafs*25	HM
					**LI‐66**	M	4	18	++	−	−	p.Val548Alafs*25	HM
XP‐LIBYA‐4	XP‐C	Yes	Yes	Al‐Zentan	**LI‐38**	F	22	24	++	+++	B.A.F	p.Arg220*	HM
XP‐LIBYA‐5	XP‐C	Yes	No	Al‐Khums	**LI‐68**	M	8	18	++	+++	1 A.K in Face	p.Val548Alafs*25	HM
XP‐LIBYA‐6	XP‐C	Yes	No	Mizda	**LI‐225**	M	Deceased at 28	24	++	+++	1 SCC (nose) + 1 A.K (Forehead)	p.Val548Alafs*25	*HM* (*deduced*)
XP‐LIBYA‐7	XP‐C	Yes	No	AL‐ Jmail	**LI‐40**	M	6	12	++	+++	−	p.Val548Alafs*25	HM
XP‐LIBYA‐8	XP‐C	Yes	No	Zleitin	**LI‐9**	F	6	36	++	+++	1 BCC with squamatization	p.Val548Alafs*25	HM
					**LI‐46**	F	6	36	++	+++	1 BCC with squamatization and 1 SCC in situ.	p.Val548Alafs*25	HM
XP‐LIBYA‐9	XP‐C	Yes	Yes	Al‐Khums	**LI‐182**	M	10	9	++	+++	−	p.Val548Alafs*25	HM
					**LI‐147**	M	13	18	++	+++	−	p.Val548Alafs*25	HM
					**LI‐179**	F	23	18	++	+++	1 invasive moderately differentiated SCC	p.Val548Alafs*25	HM
					**LI‐158**	F	13	8	++	+++	−	p.Val548Alafs*25	HM
XP‐LIBYA‐10	XP‐C	Yes	Yes	Gharyan	**LI‐13**	F	5	20	++	++	−	p.Val548Alafs*25	HM
XP‐LIBYA‐11	XP‐C	Yes	Yes	AL‐ Jmail	**LI‐190**	F	35	36	++	+++	1SCC + 1A.K	p.Val548Alafs*25	HM
					**LI‐204**	M	6	24	++	−	−	p.Val548Alafs*25	HM
					**LI‐206**	M	4	30	++	−	−	p.Val548Alafs*25	HM

Abbreviations: A.K, Actinic keratosis; B.A.F., Breast Adenofibrosis; BCC, Basal Cell Carcinoma; F, Female; HM, Homozygous; HZ, Heterozygous; M, Male; P.S., Photosensitivity; P.P., Photophobia; S.C., Skin Cancer (all located in the face); SCC, Squamous Cell Carcinoma; XP‐?, The genetic complementation group is not determined.

^a^ The age when the mother/father noticed patients' symptoms.

Photosensitivity and photophobia grades are assigned according to the criteria shown in the tables below.

**Table:** 

Photosensitivity (erythema)	
Absent	−
Pink	+
Bright red	++
Bubbles	+++

Photophobia	
Absent	−
Outdoor	+
Indoor “high light”	++
Indoor “low light”	+++

Consanguinity level among patients who participated in this study reaches 93%, which is obviously very high and could be the main drive for the expression of recessive genetic disorders such as XP.

Clinical examinations showed that XP‐C patients correspond to a relatively severe clinical form of the disease. A total of eight patients (34.7%) developed precancerous lesions or cancer (basal cell carcinoma and squamous cell carcinoma) (Table 1). Basal cell carcinoma occurred in two cases belonging to the same family.

Results of the molecular study showed that 10 patients are homozygous for the *XPC* (NC_000003.12) p.Val548Alafs*25 mutation. We also confirmed that the parents (LI‐185 & LI‐186) of one deceased patient (LI‐225) were heterozygotes for this mutation. The remaining patient (LI‐38) from Zentan (a region near to the Tunisian South borders) did not carry this mutation. To identify the mutation specific for this patient, we carried out molecular screening for two additional mutations already described in Southern Tunisian XP‐C patients (Ben Rekaya et al., 2013), considering geographical proximity and hence a likely common genetic background. Sequencing results showed the absence of the c.850G > T mutation in exon 7, yet the c.658C > T (p.Arg220X) mutation located in exon 6 has been identified at the homozygous state.

For the remaining patient, XP166, exons 6, 7, and 9 of the *XPC* gene and exon 6 of the *XPA* gene (NC_000009.12) were sequenced and showed absence of any deleterious mutations. Therefore, we targeted other pathogenic mutations that have been identified among Maghreb patients (Ben Rekaya et al., 2018; Schäfer et al., 2013): Screening for c.2048G > A (p.Arg683Gln) and c.2333 T > C (p.L778P) located in exon 22 of *ERCC2* gene (NC_000019.10) and in exon 11 of *ERCC5* gene (NC_000013.11), respectively, showed the absence of these mutations. However, a homozygous SNP (rs1052555) has been identified in exon 22 of *ERCC2* gene. This variant is classified benign according to ClinVar. Patients belonging to family XPA94, were found homozygous for the p.Arg228* mutation in *XPA* gene.

## 4 DISCUSSION

In the present study, we have conducted clinical and genetic evaluation of 23 XP Libyan patients belonging to 14 unrelated families. This study represents the first genetic characterization of XP in Libya and showed the main presence of the founder *XPC* p.Val548Alafs*25 mutation in patients exhibiting only cutaneous manifestations in addition to other mutations already described.

Clinical investigation indicated that the severity of the disease among XP‐C Libyan patients is similar to that previously described in Italy, Turkey (Gozukara et al., 2001; Mahindra et al., 2008) and patients from other North African countries, mainly Tunisia (Ben Rekaya et al., 2009; Jerbi et al., 2016). Sharing a common mutation highlights phenotype–genotype correlation.

Malignancy variability has been noted, for example, patient (LI‐208) has less symptoms at the age of 9 years when compared to patient (LI‐9) who at the age of 3 years developed one basal cell carcinomas. This could be explained by different degrees of UV‐protection and therefore reflects the involvement of socioeconomic and cultural conditions in the prognosis of XP as previously illustrated (Jerbi et al., 2016). Indeed, the installment of photoprotection precociously for LI‐208 (generally parents start the photoprotection once the diagnosis of XP is made) could be a plausible hypothetical explanation.

At the epidemiological level, although no precise data are available, its frequency is suspected to be relatively high due to the high rate of consanguinity within the Libyan population, like other neighboring Maghreb countries.

Previous studies focusing on mutational basis of *XPC* in different populations revealed no specific hot spot in the *XPC* gene. Mutation in exon 9, named p.Val548Alafs*25, however, was detected in three unrelated families from Italy, Algeria, and Morocco (Daya‐Grosjean & Sarasin, 2005; Maillard et al., 2008). This mutation has shown to be the most common mutation in Morocco with a frequency reaching 76.19% in XP Moroccan families (Senhaji et al., 2013).

Results obtained through genetic investigation of Libyan patients are very similar to other findings in patients from neighboring countries. Indeed, XPC p.Val548Alafs*25 mutation has been found to be the most frequent mutation in Tunisia and is identified in more than 50% of XP‐C patients [unpublished data]. In Algerian population, the frequency of this mutation was found to be 100% (17/17 XPC tested cases) (Bensenouci et al., 2016). In Moroccan patients, it was found to be present in 67% of cases (16 of 21) (Soufir et al., 2010). In patients from the Maghreb region, the frequency of this mutation was found to be 87% (49 of 56 XPC tested cases) (Soufir et al., 2010). Since the majority of XPC Libyan patients carry the same mutation as other XPC patients from Maghreb and Mediterranean countries, lets suggest that they all share a founder mutation with a common ancestor. Indeed, many studies have shown that the p.Val548Alafs*25 mutation was spread in North Africa, Italy, and Spain from the same ancestor. Soufir et al investigated the possibility of the existence of a founder ancestor for this mutation in the Mediterranean region using mathematical tools based on microsatellites haplotyping. The team concluded that this mutation had occurred about 1250 years ago equivalent to 50 generations back, which corresponds approximately to the time when Muslims from the Arab peninsula invaded North Africa and Europe (Soufir et al., 2010).

Furthermore, previous investigations of Tunisian XP patients have shown the presence of two founder mutations: XPC p.Val548Alafs*25 and XPA p.Arg228* (Ben Rekaya et al., 2009; Jerbi et al., 2016; Messaoud et al., 2010). These mutations have been identified in more than 90% of XP‐C and XP‐A patients. Therefore, based on these previous results, we have focused our initial efforts on screening for these founder mutations. In the present study, we identified the p.Arg228* mutation in *XPA* gene in just one family. This result reflects a common genetic background among countries from the Maghreb. Nevertheless, the hypothesis of a hot spot could not be ruled out since this variant was also described in other populations including the Japanese patients where it is described as the second common *XPA* mutation (Nishigori et al., 2019). Investigating other XP Libyan patients would tell us more about the frequency of each of these mutations and more in terms of novel mutations or variants already identified in other countries.

Our preliminary findings are extremely interesting and highly valuable in designing a simple molecular tool for early diagnosis of XP in Libyan patients and for the confirmation of clinical diagnosis.

Most importantly, results from this research prepare the ground to initiate a national screening program involving all relatives of affected individuals to identify carriers from normal homozygous among phenotypically healthy individuals. Once carriers have been identified, a precise and concrete genetic counseling can be provided for those individuals, which will help them take an informed decision before having a parental project, especially those who are involved or planning to be involved in a consanguineous marriage.

Previously, our goal was to conduct a pilot study on (i) patients to identify the genetic etiology of XP in Libya, (ii) relatives in order to set up procedures for genetic counseling and prenatal diagnosis, and (iii) a representative number of the population in order to assess the carrier frequency in the country. Unfortunately, due to political and economic challenges that faced the country during the last decade, our study was limited to the first goal.

By this study, we launch a call for decision‐makers and stakeholders in the health field to build on these findings and set a national program targeting this disease. This would certainly improve not only patients' management and families' life quality, but also positively impact on the healthcare system.

Theoretically, it seems very easy to implement a national program for cascade screening but practically, endless debates have been conducted around the experiences of different countries on genetic screening. Indeed, when we speak about population screening, many questions arise. First, we should have a clear idea about the burden of the disease in terms of prevalence and charge it impacts on the public health system. Second, we should think about the technical feasibility of the genetic testing. Third, we should carefully examine the ethical, legal, and cultural aspects related to cascade testing in order to well define how genetic testing is performed? Who can benefit of it? How should family members be contacted? etc (Newson & Humphries, 2005). Finally, we should keep in mind that besides the negative effects always glued to consanguinity, there are many positive sides that include not only socio and economic benefits but also a possible beneficial epigenetic role known as “inbreeding de‐repression” (Nebert et al., 2010). This is important to know in order to well define the priority regarding genetic testing: Are consanguineous communities more predisposed to genetic disorders and hence have a higher priority than nonconsanguineous ones?

Identification of the genetic basis of XP will, no doubt, pave the way for a better health care for XP patients. Early diagnosis of this disorder will give patients the opportunity to receive better treatment and early protection from sunlight. Implementing a national screening program will be a useful initiative for global health improvement at the national level as its application could be extended to other rare disorders that seem to be relatively frequent in Libya. Raising awareness of the civil society will be the key success point of such initiative.

## AUTHOR CONTRIBUTIONS

Najlaa Khalat was involved in patients' recruitment, molecular diagnosis, data analysis, and manuscript drafting. Olfa Messaoud was involved in manuscript drafting and revision, data analysis, and study supervision. Mariem Ben Rekaya was involved in manuscript drafting and molecular investigation. Mariem Chargui was involved in the molecular study and technical assistance. Mohamed Zghal was involved in clinical diagnosis and characterization of patients followed in Tunisia and results evaluation. Bashir Zendah and Najat Saqer were involved in clinical diagnosis and characterization for patients followed in Libya. Mourad Mokni was involved in clinical diagnosis and characterization of patients followed in Tunisia. Sonia Abdelhak and Othman A. Mohamed were involved in study design and supervision, and manuscript revision.

## FUNDING INFORMATION

This work was supported by the Tunisian Ministry of Public Health, the Ministry of Higher Education and Scientific Research (LR16IPT05) and, we would like to express our deepest gratitude to the National Authority for Scientific Research (NASR) for having funded this work in Libya.

## ETHICS STATEMENT

This study was approved by the ethical committee of Institut Pasteur de Tunis.

## Data Availability

Data available on request from the authors.
